# Aspirin and Low-Molecular Weight Heparin Combination Therapy Effectively Prevents Recurrent Miscarriage in Hyperhomocysteinemic Women

**DOI:** 10.1371/journal.pone.0074155

**Published:** 2013-09-05

**Authors:** Pratip Chakraborty, Sayani Banerjee, Piyali Saha, Shyam Sundar Nandi, Sunita Sharma, Sourendra K. Goswami, Baidyanath Chakravarty, Syed N. Kabir

**Affiliations:** 1 Department of Infertility, Institute of Reproductive Medicine, Salt Lake City, Kolkata, West Bengal, India; 2 Reproductive Biology Research, CSIR-Indian Institute of Chemical Biology, Jadavpur, Kolkata, West Bengal, India; 3 Molecular and Cell Biology, CSIR-Indian Institute of Chemical Biology, Jadavpur, Kolkata, West Bengal, India; Imperial College London, United Kingdom

## Abstract

The management of recurrent pregnancy loss (RPL) still remains a great challenge, and women with polycystic ovarian syndrome (PCOS) are at a greater risk for spontaneous abortion. Treatment with low-molecular-weight heparin (LMWH) has become an accepted treatment option for women with RPL; however, the subgroup of women, who are likely to respond to LMWH, has not been precisely identified. The present study evaluated the efficacy of LMWH with reference to PCOS and associated metabolic phenotypes including hyperhomocysteinemia (HHcy), insulin resistance (IR) and obesity. This prospective observational study was conducted at Institute of Reproductive Medicine, Kolkata, India. A total of 967 women with history of 2 or more consecutive first trimester abortions were screened and 336 were selected for the study. The selected patients were initially divided on the basis of presence or absence of PCOS, while subsequent stratification was based on HHcy, IR and/or obesity. The subjects had treatment with aspirin during one conception cycle and aspirin-LMWH combined anticoagulant therapy for the immediate next conception cycle, if the first treated cycle was unsuccessful. Pregnancy salvage was the sole outcome measure. The overall rate of pregnancy salvage following aspirin therapy was 43.15%, which was mostly represented by normohomocysteinemic women, while the salvage rate was lower in the HHcy populations irrespective of the presence or absence of PCOS, IR, or obesity. By contrast, aspirin-LMWH combined therapy could rescue 66.84% pregnancies in the aspirin-failed cases. Logistic regression analyses showed that HHcy remained a significant factor in predicting salvage rates in the PCOS, IR, and obese subpopulations controlled for other confounding factors. With regard to pregnancy salvage, combined anticoagulant therapy with aspirin and LMWH conferred added benefit to those with HHcy phenotype.

## Introduction

Recurrent pregnancy loss (RPL), traditionally defined as three or more consecutive pregnancy losses before 20^th^ week of pregnancy [1], is a surprisingly common occurrence. A number of etiologic factors have been identified for RPL. While parental chromosomal anomalies, maternal thrombophilic disorders and uterine structural anomalies have been directly associated with recurrent miscarriage, in almost 50% of cases the pathophysiology remains unknown [2]. Clinical reports document that women with polycystic ovarian syndrome (PCOS) sequelae may suffer a greater risk of spontaneous miscarriage. The prevalence of polycystic ovaries in women with recurrent miscarriage is reported to be 40–56% [3]. The cause and effect relationship between PCOS and RPL remains unknown, however, high prevalence of obesity and insulin resistance (IR) in PCOS population are postulated to be causally related [4]. But it is perplexing that even after controlling most of these factors the PCO subjects demonstrate impaired implantation and increased rate of miscarriage [3].

Pregnancy is a hypercoagulable state. Successful pregnancy outcome is highly dependent on satisfactory placental development and sustained placental function [5]. Over the last decade, evidence has accumulated to suggest that some cases of RPL and later pregnancy complications are due to an exaggerated haemostatic response during pregnancy leading to placental thrombosis and infarction [6]. Compromised placental perfusion caused by thrombosis may lead to placental infarctions and maternal complications of pregnancy [5]. Reports published during the recent past suggest that RPL is associated with an increased risk of thrombosis [5], [7]. Mild-to-moderate hyperhomocysteinemia (HHcy), a risk factor for arterial and venous thrombosis, has been suggested as a possible threat to women with habitual abortions or placental abruption. A number of studies document close association between IR and HHcy [8], with incidence of the latter being increasingly a frequent finding among PCOS women [9]. Recent evidence also suggests an association between obesity and miscarriage, while obesity appears to be a close associate of PCOS. Obesity perhaps provokes thrombosis via several mechanisms including increased activity of the coagulation cascade and decreased activity of the fibrinolytic cascade [10]. Thus, PCOS involves several confounding factors that may contribute, individually or in combination, to thrombosis and eventually lead to RPL.

In women with recurrent miscarriage and a diagnosis of antiphospholipid syndrome (APS), treatment with aspirin and heparin has been suggested to improve the pregnancy outcome, although findings from available randomized trials have been inconsistent [11], [12], [13]. It is presumed that the pathogenesis between unexplained RPL and APS-associated RPL are similar [14]. Reports also suggest the presence of inflammation and thrombosis and infarctions in the placenta and decidua of patients with pregnancy complications like RPL [15], [16]. These form the bases of anticoagulant therapy, preferably in the form of low-molecular-weight heparin (LMWH), as an accepted treatment option in several circumstances during pregnancy including unexplained RPL [14], [17]. Since the use of LMWH in the first trimester pregnancy appears to be safe for the mother and child [18], we have introduced routine use of LMWH in the management of RPL. The present study evaluates the pregnancy salvaging effects of combined aspirin-LMWH treatment for one conception cycle in a heterogeneous cohort of RPL women, who had unsuccessful outcomes following aspirin therapy during their last conception. The objective is to identify if any specific subgroup of RPL are likely to benefit from the use of LMWH.

## Methods

### Patient Selection and Study Design

**Figure 1:**
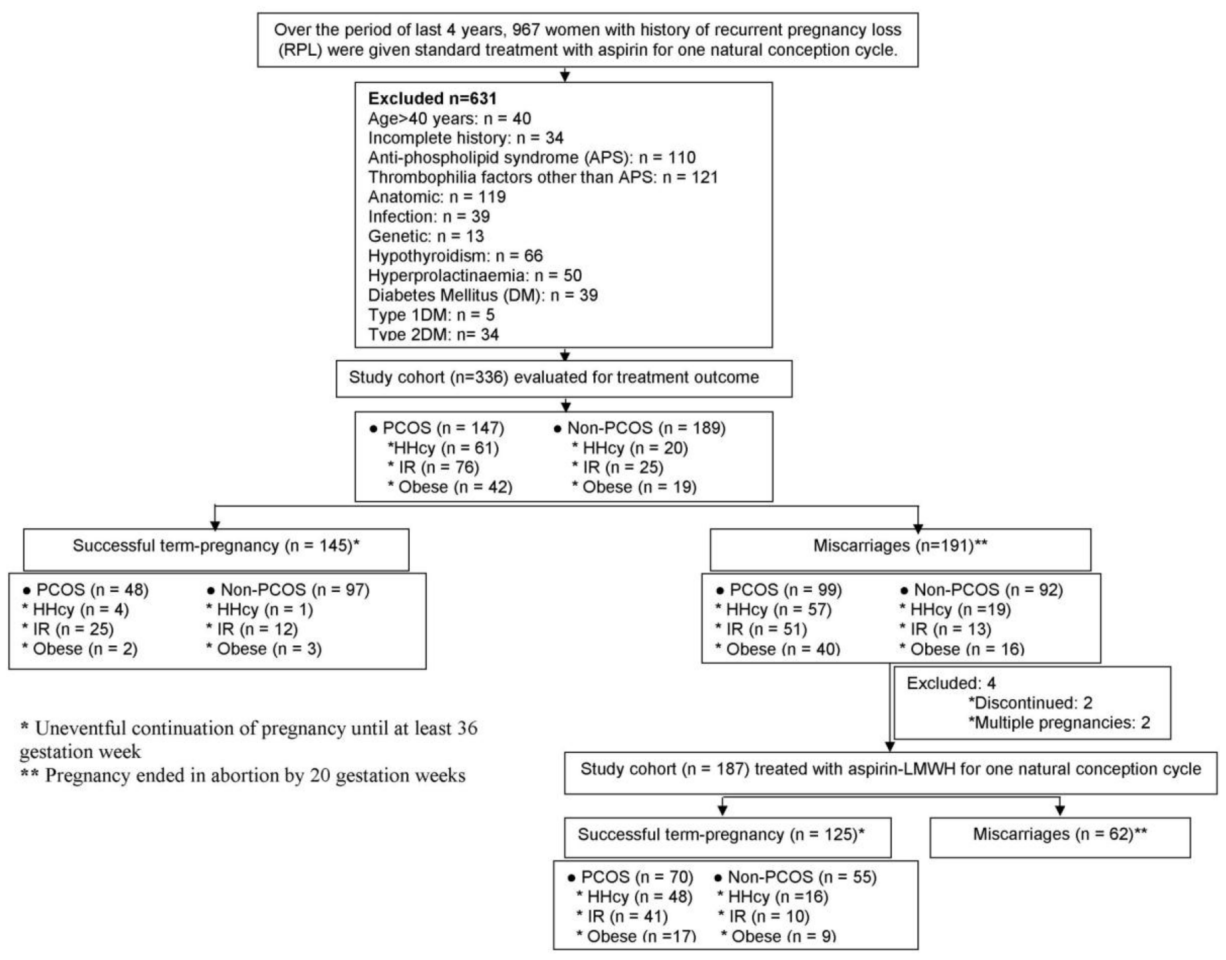
Flow chart for patient selection, phenotype distribution, treatment and pregnancy outcome.

This prospective observational study was conducted at Institute of Reproductive Medicine, a referral infertility clinic in Kolkata, India, from January 2008 through December 2011. The study protocol was approved by the Institutional Ethics Committee of Institute of Reproductive Medicine, Kokata governed by Indian Council of Medical Research, New Delhi (Ref. No. IRM/IEC/BNC-IHP-37/15-09-2007). Written informed consent was obtained from all the participants prior to enrolling in the study. The subjects recruited for the present investigation were selected from a total of 967 women with history of RPL, who were treated with low dose acetylsalicylacid (ASA) during their last spontaneous pregnancy. The selection was based on the exclusion criteria: age >40 years, ultrasonography (USG)-documented uterine anatomical anomalies, chromosomal defects as evaluated by peripheral blood karyotyping of both partners, hypothyroidism, hyperprolactinemia, diabetes mellitus, positive tests for APS (lupus anticoagulant and anticardiolipin antibodies), infections including Toxoplasma gondii, herpes simplex virus, and cytomegalovirus, and unavailability of relevant clinical and laboratory data. Three hundred thirty-six women met the inclusion criteria. Based on characteristic phenotypes, the patients were stratified: the presence or absence of PCOS was the initial dividing criteria, while subsequent stratification was based on plasma levels of homocysteine (Hcy), IR, and body mass index (BMI). The pregnancy outcome following ASA treatment was evaluated with respect to their specific phenotypes. There were 191 women who had failed pregnancy following ASA therapy. Of these, 1 non-PCOS woman and another PCOS woman with obesity were not interested in further achieving pregnancy, while 186 women who were planning to conceive and 3 women who were spontaneously pregnant with gestation age of less than 6 weeks finally formed the study cohort for ASA-LMWH combined anticoagulant therapy. Subsequently 2 PCOS women with obesity were excluded from the study due to multiple pregnancies. Thus, 187 women finally received LMWH (Fragmin, Pfizer, US) at a prophylactic dose of 2500 IU sc everyday in concomitant with ASA 5 mg/day since foetal cardiac activity was observed by USG and continuing up to 12 weeks of gestation. Like in their last unsuccessfully-treated pregnancies, all patients also received luteal support in the form of intravaginal micronised progesterone (100 mg, twice daily), vitamin B12 and folic acid (10 mg/day) as a part of antenatal care, and metformin (500 mg/twice a day), for those diagnosed with IR, continuing until term. The PCOS subjects had no specific dietary therapy other than the lifestyle modification program with dietary and exercise interventions that are routinely recommended to improve the PCOS-associated fertility and metabolic complications. The solitary outcome measure was the rate of pregnancy salvage, which was evaluated with respect to Hcy level, IR status and obesity separately in both treatment populations. Salvaged pregnancy was defined as an uneventful continuation of pregnancy until at least 36 weeks. Delivery of babies was effected by caesarean section performed usually at 37^th^ week, and occasionally at 36^th^ week, if the situation so demanded. The flow chart for patient selection and distribution of different phenotypes among the 2 treatment cohorts are schematically presented in Figure 1.

### Diagnosis of PCOS

The diagnosis of PCOS was done in accordance with the Rotterdam criteria [19]. According to these criteria, PCOS was diagnosed if at least two of the following criteria were present: oligo/amenorrhoea, clinical or biochemical hyperandrogenism and PCO on USG. Other causes of hyperandrogenism (prolactinoma, congenital adrenal hyperplasia, Cushing Syndrome and virilizing ovarian/adrenal tumors) were excluded.

### Laboratory Evaluation

The laboratory analyses used the following assays to measure the endocrine and biochemical parameters. FSH, LH, testosterone (total), insulin and homocysteine (Hcy) were assayed in serum with automated chemiluminescence assay system using an Immulite® platform (Diagnostic Products Corporation, Los Angeles, CA, USA). The intra- and inter-assay coefficients of variation were <10% for all assays performed. Hcy level of 12 µmol/L was set as the cut-off limit for HHcy. Glucose levels were measured using VITROS dry Chemistry System® (Ortho-Clinical Diagnostics, Strasbourg, France). IR was assessed using the homeostatic model assessment 2 (HOMA2-IR: (fasting insulin×fasting glucose)/22.5) calculated by using the Oxford Diabetes Trials Unit calculator (http://www.dtu.ox.ac.uk; University of Oxford, UK). Patients with HOMA2-IR greater than 2.1 were classified as IR [20]. The BMI was determined using the formula: BMI = weight/height^2^ (kg/m^2^). Patients were classified into obese (BMI ≥30 kg/m2) and non-obese (BMI <30 kg/m2) according to the World Health Organization classification system for obesity [21].

### Statistics

Baseline characteristics of the two different RPL subgroups were analyzed by analysis of variance. Proportions were compared using the chi-square test. A logistical regression model was used to assess the effect of HHcy, PCOS and other confounding factors on pregnancy salvage. Statistical Package for Social Sciences version 17.0 (SPSS Inc, Chicago, Illinois, USA) was used for statistical analysis. Data are presented as mean ± SD. *P*-value <0.05 was considered statistically different.

## Results

### ASA Therapy

The baseline characteristics of the patients and overall pregnancy outcomes following treatment with aspirin in combination with or without LMWH are summarized in Table 1. Overall, the prevalence of PCOS was 43.75% (n = 147) in the selected cohort treated with aspirin alone. The 2 subpopulations with and without PCOS differed in respects of plasma Hcy levels, HOMA2-IR, and BMI that were significantly (*P*<0.0001) higher in the PCOS subgroup. Amongst women with PCOS there were also higher (*P*<0.0001) incidences of HHcy (41.49%), IR (51.70%), and obesity (28.57%) than those of without PCOS.

**Table 1:** Clinical and biochemical characteristics and pregnancy outcomes in the aspirin, and aspirin+LMWH-treated populations.

Variables	Aspirin			Aspirin+LMWH		
	Total (n = 336)	PCOS (n = 147)	Non-PCOS (n = 189)	Total (n = 187)	PCOS (n = 96)	Non-PCOS (n = 91)
		43.75%	56.25%		51.33%	48.66%
Age (years)	33.02±3.21	32.98±4.09	33.05±4.66	33.63±5.94	33.46±4.20	33.80±5.94
Previous no. of miscarriages	2.51±0.93	2.49±0.98	2.52±0.83	3.41±0.71	3.48±0.72	3.33±0.76
Plasma Hcy (µmol/l)	10.59±5.61^a^	13.29±3.13^b^	8.49±2.76^c^	11.21±3.50^a^	13.32±3.17^b^	8.98±3.50^c^
HOMA-2 IR	1.97±1.11^a^	2.43±0.98^b^	1.61±1.16^c^	2.06±1.02^a^	2.28±1.12^b^	1.82±1.07^c^
BMI (kg/m^2^)	23.38±3.72^a^	26.18±2.69^b^	23.75±2.09^a^	24.97±2.71^a^	26.22±2.62^b^	23.65±2.47^c^
HHcy (%)	24.10^a^	41.49^b^	10.58^c^	40.64^a^	59.37^b^	20.87^c^
IR (%)	30.05^a^	51.70^b^	13.22^c^	34.22^a^	53.12^b^	14.28^c^
Obesity (%)	18.15^a^	28.57^b^	10.05^c^	28.34^a^	38.54^b^	17.58^c^
Pregnancy Salvage (%)	43.15^a^	32.65^b^	51.32^a^	66.84	72.91	60.43

Values are expressed as mean ± S.D.

HHcy: hyperhomocysteinemia (Hcy>12 µmol/l); IR: insulin resistance (HOMA2-IR>2.1); NIR: non-insulin resistance; HOMA-2-IR: homeostatic model assessment 2-insulin resistance; obesity: BMI ≥30 kg/m^2^; pregnancy salvage: uneventful continuation of pregnancy at least until 36^th^ week.

a, b, c Values with different superscripts in a row under specific treatment population differ significantly.

a *vs.* b: P<0.037; b *vs.* c: P<0.002; a *vs.* c: P<0.016.

The overall rate of pregnancy salvage in the total cohort was 43.15%, and there was no incidence of preeclampsia-like symptom or preterm labor. The results of univariate analysis showed that the rate of salvage in the PCOS subpopulation (32.65%) was significantly lower (*P*<0.0009) than that of non-PCOS (51.32%) (Table 1). The rate of salvage in relation to each of the confounding factors adjusted for the remaining factors, and corresponding odds ratio (OR) resulting from multivariate regression analysis are shown in table 2. Except HHcy, which was negatively associated with pregnancy salvage (OR, 0.27; 95% confidence interval (CI), 0.08–0.80), none of obesity, IR and PCOS was found to have any association with treatment outcome.

**Table 2:** Raw (%) and adjusted (OR) rates of pregnancy salvage following aspirin treatment.

Factors		N	% of pregnancy salvage (n)	OR (95%CI)	p-value
Total Cohort		336			
PCOS	No	189	51.32 (97)	1	
	Yes	147	32.65 (48)	1.57 (0.83–2.98)	0.16
HHcy	No (≤12 µmol/L)	255	54.90 (140)	1	
	Yes (≥12 µmol/L)	81	6.17 (5)	0.27 (0.08–0.80)	0.02
IR	No (HOMA2-IR≤2.1)	235	45.95 (108)	1	
	Yes (HOMA2-IR≥2.1)	101	36.63 (37)	0.75 (0.56–1.01)	0.09
Obesity	No (BMI≤30 kg/m^2^)	275	50.90 (140)	1	
	Yes (BMI≥30 kg/m^2^)	61	8.19 (5)	0.94 (0.85–1.04)	0.24

HHcy: hyperhomocysteinemia (Hcy>12 µmol/l); IR: insulin resistance (HOMA2-IR>2.1); obesity: BMI ≥30 kg/m^2^; OR: odds ratio; CI: confidence interval.

Column percentages presented for univariate analysis.

### ASA-LMWH Combined Therapy

The selected cohort receiving aspirin-LMWH combined therapy included 51.33% women with PCOS. The 2 subpopulations with and without PCOS differed in respects of HOMA2-IR (*P*<0.0004), plasma Hcy level (*P*<0.0001), and BMI (*P*<0.0001) that were significantly higher in the PCOS subgroup. The overall incidences of HHcy, IR, and obesity in the total cohort were 40.64%, 34.22%, and 28.34%, respectively; while the incidences of all three phenotypes were significantly higher (*P*<0.002) in the PCOS population (HHcy: 59.37%, IR: 53.12%; obesity: 38.54%) as compared with those of non-PCOS population (HHcy: 20.87%, IR: 14.28%; obesity: 17.58%). Overall, pregnancy was salvaged in 66.84% of the total cohort (Table 1) without any incidence of preeclampsia-like syndrome or preterm labor.

Univariate analysis demonstrated the salvage rate was more in the PCOS population (72.91%) compared to the non-PCOS population (60.43%), but the difference was not statistically significant. Multivariate logistic regression analysis showed that neither PCOS status, nor obesity, but HHcy (OR, 1.55; CI, 1.29–1.88) and IR (OR, 1.20; 95% CI, 0.82–1.43) had significant effects on salvage (Table 3). Analyses in the IR, obese and PCOS subpopulations showed that HHcy remained a significant factor for differential salvage rates in the IR group (OR, 1.56; 95% CI, 1.05–2.14), obese group (OR, 1.25; CI, 1.07–1.39) and PCOS group (OR, 1.12; 95% CI, 1.02–1.57), controlling for other confounding factors.

**Table 3:** Raw (%) and adjusted (OR) rates of pregnancy salvage following combined anti-coagulant therapy with aspirin and LMWH treatment.

Factors		N	% of pregnancy salvage (n)	OR (95%CI)	p-value
Total Cohort		187			
PCOS	No	91	60.43 (55)	1	
	Yes	96	72.91 (70)	0.78 (0.63–1.57)	0.33
HHcy	No (≤12 µmol/L)	111	54.95 (61)	1	
	Yes (≥12 µmol/L)	76	84.21 (64)	1.55 (1.29–1.88)	0.0001
IR	No (HOMA2-IR≤2.1)	123	60.16 (74)	1	
	Yes (HOMA2-IR≥2.1)	64	79.68 (51)	1.20 (0.82–1.43)	0.02
Obesity	No (BMI≤30 kg/m^2^)	134	73.88 (99)	1	
	Yes (BMI≥30 kg/m^2^)	53	49.05 (26)	0.91 (0.53–1.56)	0.72

HHcy: hyperhomocysteinemia (Hcy>12 µmol/l); IR: insulin resistance (HOMA2-IR>2.1); obesity: BMI ≥30 kg/m^2^; OR: odds ratio; CI: confidence interval.

Column percentages presented for univariate analysis.

## Discussion

With a goal to improving the rate of live births, aspirin and LMWH have been tried for women with unexplained RPL, but the reported success rates vary widely between the studies [12], [22]. In our clinic, it was a routine practice to use prophylactic doses of aspirin along with folic acid and vitamin B12 for the management of pregnancy in women with bad obstetric history. Since LMWH does not cross the placenta [23] and appears to be safe for mothers and fetus at all stages of pregnancy [24], we have introduced prophylactic use of combined aspirin-LMWH therapy since recent past. The present communication reports the outcomes of aspirin-LMWH combined anti-coagulant therapy in a group of RPL women who did not benefit from treatment with aspirin during their last conception cycle.

Women with RPL may encompass a wide range of phenotypes [25]. During the last 15 years, the list of candidate causes for RPL has grown rapidly. Recent evidence suggests a causal association between HHcy and RPL [26]; and mild-to-moderate degree of HHcy leads to a 3-fold increase in the risk of early pregnancy loss [27]. On the other hand, obesity and IR have been implicated as individual risk factors for RPL [28], while HHcy, IR, and obesity are very common associates of PCOS [8], [29]. Here we report two studies involving two separate populations: one treated with aspirin, and the other treated with aspirin-LMWH together. Both populations were represented by PCOS and non-PCOS women with occurrence of HHcy, IR, or obesity, which were the phenotypes the further stratifications based upon. Pregnancy salvage was evaluated separately in each population with respect to different phenotypes by fitting logistic regression models.

There is no international consensus regarding the establishment of a cut-off point for HHcy. Hcy values of between 10 and 12 µmol/L in pregnant individuals are considered borderline elevated [30]. Fasting plasma total Hcy value of 12 µmol/L was therefore set as the cut-off limit for the present investigation. Evaluation of the impact of different degrees of obesity on the risk of recurrent miscarriage demonstrated that not a mild increase in the BMI (25.0–29.9 kg/m^2^), but obesity characterized by BMI >30 kg/m^2^, increases the risk of miscarriage [31]. We therefore set BMI value of 30 kg/m^2^ as the cut-off to differentiate obese from non-obese population.

This is a prospective study but not ideally designed since the plan for aspirin-LMWH combined therapy was conceived after the results of treatment with aspirin was mostly available. In neither of the studies proper placebo-controlled group was maintained. Also, co-treatments with folic acid, vitamin B12, and progesterone, which we routinely use towards antenatal care in normal as well as high risk pregnancies, could not be avoided because of ethical objection. Thus, in addition to the anticoagulants, both study populations were exposed to multiple therapies which made it difficult to assign the salvage precisely to one component of the therapies. However, the data gathered from these studies do provide some important messages.

Our data seem to show that aspirin-LMWH combined therapy significantly prevented RPL in women with HHcy phenotype, who were not benefitted from aspirin treatment alone during their last conception cycle. Of importance to note that the incidence of obesity and HHcy were significantly higher among women who did not respond to aspirin but subsequently responded to aspirin-LMWH combined therapy.

The number of prior pregnancy losses is reported to impact one’s chance to carry next pregnancy to term [32]. The aspirin-treated group did not comply with the classical definition of RPL as there were 98 out of 336 patients with 2 previous miscarriages; while the combined therapy group met the criterion of ≥3 previous miscarriages that classically defines RPL. The different subgroups in each population, however, were statistically identical with respect to the mean number of prior pregnancy losses. We enrolled women up to 6 weeks of gestation age in the ASA-LMWH combined group that may question the possibility of skewing the data in a negative direction for aspirin. This possibility may however be ruled out because there were only 3 women who were enrolled between 5.0–5.5 weeks of gestation, and incidentally all 3 women belonged to the normohomocysteinemic subpopulation of non-PCOS group. Moreover, anticoagulant therapy was initiated from 6^th^ week of pregnancy after USG confirmation of fetal cardiac activity.

Various interventions have been suggested for the management of RPL; however, no established effective treatment options are as yet identified. Although the findings from available randomized trials have been inconsistent [12], [13], many authorities consider that RPL with diagnosis of heritable or acquired hemophilias do benefit from antithrombotic therapy using aspirin and LMWH [33], [34]. The proposition that women with unexplained RPL might benefit from anti-coagulant therapy is based on the presumption that in many women thrombotic damage and increased generation of thrombin may not be identifiable by routine laboratory investigations, although a thrombophilic maternal phenotype commonly underlies; and RPL may be attributed to localized thrombosis in decidual vessels [35]. Perhaps this presumption holds specifically valid under HHcy condition, which has long been described as a risk factor for arterial and venous thrombosis, and suggested as a possible risk factor for placental abruption or infarction and impaired chorionic villous vascularization [36]. HHcy may lead to early damage to decidual or chorionic vessels that may cause disturbed implantation of the conceptus. Tulpalla et al. (1991) reported that women with history of RPL have shift in the thromboxane A2: prostaglandin I_2_ ratio in favour of the former [37], which may lead to vasospasm and platelet aggregation in the early trophoblast and development of microthrombi and placental necrosis that are often seen in the placentas of women with habitual abortion [38]. It is possible that anticoagulant therapy corrects these biochemical abnormalities.

Many observational studies represent a significant body of data that advocate use of LMWH in unexplained RPL [18], [39], while many other trial data fail to substantiate any beneficial role of aspirin in combination with LMWH [22], [40]. Variation in the reported outcomes may be due to differences in the characteristic phenotypes of the study population. In a recently published randomized controlled trial, Kaandorp et al (2010) reported that neither aspirin alone nor combined with a LMWH preparation, nadroparin, improved the live-birth rate, as compared with placebo, among women with unexplained recurrent miscarriage [40]. It may be significant to note that along with the known and potential causes that were accounted to diagnose unexplained RPL, the reported study also included HHcy as the excluding criterion. Therefore, the role of LMWH in miscarriages of unexplained etiology may be questioned, but we strongly feel that the outcome of the trial conducted by Kaandorp et al (2010) would have been different if HHcy women were not excluded.

The treatment with LMWH is perhaps useful in preventing RPL, but the timing of therapy is yet to be optimized [16]. Based on the assumption that the main therapeutic effect of LMWH is the inhibition of thrombosis, some advocate the therapy in the first trimester when the platelets become thrombophilic [41], [42], while the others who consider its main role in improving implantation, recommend initiation of therapy since the time of ovulation, or even earlier [42]. We, however, started LMWH treatment only after ultrasound detection of pregnancy, and as against the conventional protocol of continuing anticoagulant regimen through 32–34 weeks, we continued LMWH treatment till 12 weeks of gestation. Trophoblastic proliferation and maternal blood flow into the chorio-decidual space starts early in pregnancy and is completed by 10 weeks [43]. Risk of placental ischemia and thrombosis is maximum during this period and wanes thereafter [44]. This remains the basis of restricting the LMWH treatment till 12 weeks of gestation. Moreover, restricted use of anticoagulants may reduce the risk of fracture and osteoporosis and also avoid monitoring complexity of patients coming from far off places – especially in our set-up where a systematic network of monitoring is not available. However, cessation of LMWH therapy after 12 weeks of pregnancy was not confronted with any undesired consequences.

The present report thus represents a preliminary study with several limitations. This is an observational study not supported by appropriate untreated controls. Moreover, the way the patients were recruited for combination therapy (those unsuccessfully treated with aspirin in their last conception cycle) might have filtering effect that made the two treatment populations not exchangeable and comparable in certain respects. Nonetheless, significantly improved rate of pregnancy salvage by combined aspirin and LMWH therapy in subjects with HHcy phenotype suggest the protective effects of combined anticoagulant therapy. It is relevant in this context that controversy still prevails on the issues whether addition of aspirin to heparin therapy has any added benefit, and also the type of heparin, that is, unfractionated versus LMWH, to be preferred [42]. The recent realization that LMWH, like unfractionated heparin (UFH), also risks thrombocytopenia, and UFH better prevents complement activation prompts some investigators to prefer UFH to LMWH [42]. There are, however, other recent reports that claim better safety profile of LMWH with reference to incidence of thrombocytopenia [45] and cytoprotection [46]. These issues will perhaps be resolved once more data are generated through controlled studies.

In conclusion, combined anticoagulant therapy with aspirin and LMWH seem to confer added benefit to those with HHcy phenotypes. The present study should, however, be confirmed in a randomized trial with control arms in a large cohort. We are about to initiate such a multi-centric trial. Until the trial outcomes are available, the observation provided herein may be merely viewed as a prelude to what the future holds.
